# “I’ve Heard of It, Yes, but I Can’t Remember What Exactly It Was”—A Qualitative Study on Awareness, Knowledge, and Use of the UV Index

**DOI:** 10.3390/ijerph18041615

**Published:** 2021-02-08

**Authors:** Katharina Diehl, Tatiana Görig, Charlotte Jansen, Maike Carola Hruby, Annette B. Pfahlberg, Olaf Gefeller

**Affiliations:** 1 Mannheim Institute of Public Health, Social and Preventive Medicine, Medical Faculty Mannheim, Heidelberg University, 68167 Mannheim, Germany; katharina.diehl@medma.uni-heidelberg.de (K.D.); tatiana.goerig@medma.uni-heidelberg.de (T.G.); charlotte.jansen@medma.uni-heidelberg.de (C.J.); hruby@stud.uni-heidelberg.de (M.C.H.); 2 Department of Medical Informatics, Biometry and Epidemiology, Friedrich-Alexander-University of Erlangen-Nuremberg, 91054 Erlangen, Germany; annette.pfahlberg@fau.de

**Keywords:** UV index, pharmacy, pharmacists, counselling, education, sun protection, sun protection factor, prevention, skin cancer

## Abstract

Pharmacists and pharmaceutical technicians play an important role in counselling customers regarding sunscreen use and sun protection measures. A potentially helpful tool that can be used during counselling is the ultraviolet index (UVI), which informs individuals when and what sun protection measures are needed at a specific place and time. Our aim in this qualitative study was to explore awareness, knowledge, and use of the UVI during counselling in pharmacies. We used semi-structured interviews with pharmacists and pharmaceutical technicians (*n* = 20) to answer our research questions. Interviews were audiotaped, transcribed verbatim, and analyzed using qualitative content analysis. During the interviews pharmacists and pharmaceutical technicians revealed a lot of uncertainty and lack of knowledge regarding the UVI. Eight professionals were able to give a correct definition of UVI. Amongst others, the UVI was confused with sun protection factor. Overall, the UVI was hardly used during the counselling of customers. The UVI was developed to provide guidance when which type of sun protection is required to avoid detrimental effects of ultraviolet radiation. For effective implementation, both the general population and health professionals (e.g., pharmacists) have to increase their knowledge about the UVI. This would strengthen its use during professional counselling in pharmacies and may help to reduce the incidence of skin cancer over the long term.

## 1. Introduction

During the past decades, there has been a rise in incidence rates of skin cancer in Caucasian populations worldwide [1,2,3,4,5]. The main environmental risk factor for the most common skin cancers (i.e., cutaneous melanoma and keratinocyte carcinomas) is solar ultraviolet (UV) radiation [6,7,8]. Therefore, to prevent skin cancer, it is important to promote sun protection behaviours.

One important measure regarding sun protection is sunscreen use [9,10,11]. A representative study in Germany showed that more than four out of ten people (43.8%) use sunscreen always or often on a sunny summer day [12]. In Germany, as in many other countries, sunscreen can be bought in supermarkets, drugstores, and pharmacies. An advantage of buying sunscreen in pharmacies is personalized counselling, which is promoted by some pharmacies online or on posters in shop windows. Additionally, pharmacists are generally viewed as credible [13]; a worldwide survey reported that 86% of respondents completely or predominantly trust pharmacists [14]. The German Pharmacist Newspaper wrote in 2018 that “The pharmacy offers a good point of contact for information and advice on skin cancer prevention” [15]. Further, Tucker and Duffy [16] reported that counselling on sun protection measures is an important health promotional activity in pharmacies. In a survey among 3129 parents of 3- to 6-year-old children in Germany, nearly one-quarter of the respondents (22.9%) stated that they had been advised on sun protection by pharmacists and/or physicians [17].

One measure that may help health professionals, such as pharmacists and pharmaceutical technicians, to provide guidance for customers regarding the situations in which sun protection is necessary is the ultraviolet index (UVI). The UVI is a simple internationally standardized quantity capturing the intensity of UV radiation triggering a sunburn on human skin at a specific place and time [18]. More specifically, the UVI uses the concept of erythemally weighted solar UV irradiance to assess the potential of UV radiation to induce erythema (sunburn) as an acute harmful photobiological effect in human skin [19,20]. The daily UVI is defined as the highest 30-min moving average of measurements of UV spectra weighted with the erythema reference action spectrum of the International Commission on Illumination [21,22].

The UVI was originally developed by Canadian scientists in 1992, and adopted in a slightly modified version by the World Health Organization (WHO), World Meteorological Organization, International Commission on Non-Ionizing Radiation Protection, and United Nations Environment Programme in 1994 [23,24]. In 2002, the WHO and its partner organizations published a practical guide providing information on how the concept of the UVI could be extended to serve as a public awareness tool [25]. Regular reviews and adjustments of the UVI concept have taken place since then [26,27].

The purpose of the UVI is to facilitate individuals to protect themselves from natural UV radiation by getting information about the intensity level of actual UV radiation in their region. For applying sun-protective measures at the right time it is essential to have access to the information when the right time actually is since humans lack a sensory organ for UV radiation and underestimate the intensity of UV radiation during spring and early summer when concluding intensity of UV radiation solely from the temperature level during this time of the year. In its practical guide for the public, the WHO recommended sun protection measures connected to different levels of the UVI [25]. Therefor the unitless UVI, always given as an integer value, is divided into five categories: low (values 0–2), moderate (3–5), high (6–7), very high (8–10), and extreme (11+). A low value means that sun protection is only needed for individuals with very light skin during an extended outdoor stay [28]. Starting from moderate values, sun protection measures including sunscreen use (and re-application), sunglasses, protective clothing, and staying in the shade (especially around solar noon) are recommended [25]. For very high and extreme values, all of the abovementioned measures are recommended in addition to seeking shade all day and avoiding outdoor stays during peak UV hours around noon.

Based on the information provided, the UVI seems to be a feasible instrument that could be included in pharmacy counselling on sun protection, even though the UVI is not exclusively related to sunscreens and their sun protection factors but rather to more general sun-protective recommendations connected to different levels of the UVI partly depending on skin type [25,29]. General population studies in the past have, however, shown that knowledge on the UVI is deficient [30,31]. Therefore, by using semi-structured interviews, we aimed to explore to what extent pharmacists and pharmaceutical technicians are informed about the UVI and whether they incorporate their knowledge into counselling their customers on sun protection.

## 2. Materials and Methods

We conducted a qualitative study among pharmacists and pharmaceutical technicians to explore their knowledge of sun protection measures and their perceived needs for properly counselling customers on sun protection. The focus of this manuscript is on awareness and understanding of the UVI and its use in counselling with customers.

### 2.1. Study Population

The participants were recruited from the Rhine-Neckar metropolitan region (Southwestern Germany) via invitation letters that were sent to all pharmacies in the area and via direct contact in pharmacies. Addresses of pharmacies were collected from the yellow pages directory. Eligible for participation were pharmacists and pharmaceutical technicians. We included those who were interested in the study until we felt that, with an increasing number of participants, the information gain of additional interviews decreased (theoretical saturation). This means that additional interviews did not reveal new information beyond the already identified aspects. Our saturation point was reached at about interview 17 and we stopped data collection by including *n* = 20 pharmacists and pharmaceutical technicians.

### 2.2. Data Collection

To collect our data, we used semi-structured, face-to-face interviews with open-ended questions (see Supplementary File). This manuscript includes analysis on awareness and, understanding of the UVI as well as knowledge of the UVI metrics (general values, values in the home region, values at which sun protection is recommended), and use of the UVI during counselling customers about sun protection).

All interviews were conducted between May and December 2019 by the same person (MCH, female medical student and graduated nurse), who was extensively trained in advance by the first author (KD), who is experienced in qualitative interviewing. MCH conducted two pretest interviews to pilot the semi-structured guide. The practicability of the interview guide was good resulting in a fluent interview process and understandability of questions was assumed as good because no queries regarding questions and terms used occurred, the pretest interviews were included in the analysis. Interviews lasted 35 min on average (min: 25 min, max: 55 min). For the interviews, participants suggested locations where they felt comfortable talking. No third parties were present during the interviews. All interviews were audiotaped (Olympus WS-853) and transcribed verbatim by MCH and CJ. Prior to the interviews, participants were informed about the study’s procedure and data protection. All participants provided written consent to participate in the study. They received a €20 gift voucher as reimbursement for their time. Approval was obtained from the Ethics Committee II of the Medical Faculty Mannheim, Heidelberg University on 26 February 2019 (number 2019-1154N).

### 2.3. Data Analysis

We used qualitative content analysis following Mayring [32] to analyze the transcripts. We systemized the data by identifying categories and subcategories (i.e., common themes within the interviews). We developed an initial set of main codes based on the semi-structured interview guide. These codes were further refined during the coding process. We coded the data using the program MAXQDA (VERBI Software GmbH, Berlin, Germany). The interview passages on the UVI were independently coded by two researchers (CJ and AE). The kappa value for consensus, following Brennan and Prediger [33], was 0.87. Disagreements were discussed and resolved by consensus in each case. Additionally, we quantified our qualitative results to provide an impression of the frequency of potential knowledge deficits.

## 3. Results

In total, 20 German pharmacy workers participated in the study. On average, they were 47.5 years old (SD = 14.9 Min = 23, Max = 69). Sixteen women (80%) and four men (20%) were interviewed. All participants completed vocational education in Germany (50% completed vocational training and 50% master’s degree or doctorate). Participants all graduated between 1980 and 2019, and 40% had a career break at some point after graduation (up to three years). Four participants were managers or heads of a pharmacy (20%), six were salaried pharmacists (30%) and ten were pharmaceutical technicians (50%). The majority worked full-time (60%). The pharmacies were mostly located in urban areas (75%) and employed between two and 25 people (M = 12.30, SD = 8.81). Three-quarters of the participants stated that they had previously taken part in advanced training for skin and sun protection. Table 1 provides detailed information on each participant to allow the reader to assess the relevance of findings based on the individual participant’s situation as suggested by the consolidated criteria for reporting qualitative research (COREQ, [34]).

### 3.1. Awareness of the Term “UV Index”

**Figure 1:**
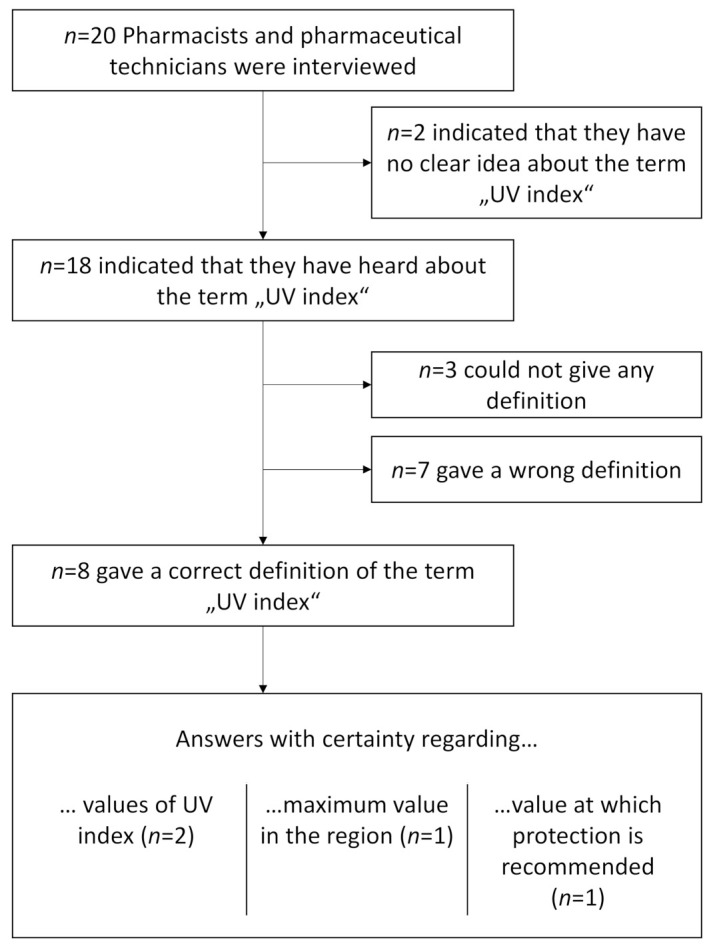
Flowchart of participants regarding answers to different aspects of the UV index.

Out of 20 participants, 18 stated they had heard the term “UV index” (Figure 1). Two (P19 and P20) admitted they had never heard this term. However, among those who indicated they were aware of the UVI, some mentioned in the same sentence that they probably would not be able to provide a definition. For instance, P01 stated “*I’ve heard of it, yes, but I can’t remember what exactly it was,*” P05 stated “*UV index, yes, but I can’t tell you exactly what it is now (laughing),*” and P09 stated, “*Yes, although I can’t remember in which context I’ve heard of it (laughing).*”

### 3.2. Understanding the Term “UV Index”

Of the 18 participants who stated they had heard the term “UV index” before, eight (40%) were able to give a correct definition (P02, P06, P07, P10, P12, P13, P17, P18). Here, an answer was coded as the correct definition of UVI, when the participants knew at least that UVI describes the strength of UV radiation. Specific technical knowledge of the definition of the UVI was not necessary to qualify for being coded as correct. Among the other ten participants, three did not give any definition (P01, P14, P16), while seven gave incorrect definitions (P03, P04, P05, P08, P09, P11, P15; Table 2).

### 3.3. Values of the UV Index

Of the eight participants who gave a correct definition for the UVI, five commented on the values of the UVI (P02, P06, P10, P12, P13), while three did not know anything about the UVI metrics (P07, P17, P18). As a correct definition, we considered a range of 0/1 to 11/12. However, among those who reported the potential values of the UVI, we found, besides wrong definitions, uncertainties in answering this question (P06, P12, P13; Table 3).

Regarding maximum values that can be reached in the Rhine-Neckar region (i.e., 9), five participants provided answers (P02, P06, P10, P12, P13); however, these answers were mostly characterized by uncertainties and vague estimates. This was also true for three out of four answers regarding the UVI value at which protective measures are recommended (P6, P10, P13). Following official recommendations, regular sun protection measures including sunscreen use should start from moderate values (i.e., 3–5) [21].

### 3.4. Counselling Situations

Participants who could define the term “UV index” reported they rarely used the term when counselling their customers on sun protection (e.g., P13: “*I don’t address it [the UV index] much during counselling*”). For instance, P12 said “*Um…well, I share it but I don’t mention the term ‘UV index.’ Well, it might happen, but I’d rather translate it for the customer.*” They further reported that, during counselling, they adapt their knowledge on the UVI by asking for what occasion the customer needed a sunscreen product (e.g., to use it on vacation or for everyday use; P02, P06, P07).

P12 reported that the term “sun protection factor” is a more commonly recognized term than “UV index,” stating “*I can tell you a lot more about sun protection factor, and people also know a lot more about it*.” Additionally, the sun protection factor (SPF) seemed to be an aspect that was more frequently addressed in counselling. For example, P06 answered a question regarding whether customers had ever asked him about the UVI by stating, “*I don’t think about UV index, but about sun protection factor.*”

## 4. Discussion

We used a qualitative approach to explore awareness, knowledge, and use of the UVI in a group of pharmacists and pharmaceutical technicians. These professionals play an important role in counselling customers regarding sun protection behaviours. Many people go to pharmacies with the purpose of buying sunscreen and hope to receive good advice from the pharmacists and pharmaceutical technicians. However, we identified severe deficits in this group regarding knowledge about the UVI, although the UVI might be a helpful tool during counselling.

There are only a few previous studies that focused on pharmacists’ knowledge and counselling regarding sun protection, although two-thirds of pharmacists in Bavaria (Germany) stated that pharmacies are a particularly suitable place for information on skin cancer prevention [35]. A recent study showed that pharmacists’ advice can definitely play a role in customers’ choices regarding sunscreen products [36], however, studies in Arizona (USA) and Iran showed knowledge deficits among pharmacists [37,38]. The study in Arizona reported that about 30% of pharmacists felt uncomfortable counselling on sun safety when asked by customers [37]. In addition, their knowledge was more dependent on their number of years in practice and having a relative with skin cancer than on training [37]. However, an older study from the US showed that training increased knowledge, self-rated expertise, and the proportion of patients counselled [13,39].

We found a lot of uncertainty regarding the definition of the term “UV index” among pharmacists and pharmaceutical technicians. In our sample, the majority of participants reported to have received advanced training on sun protection; however, a quarter (5 of 20) of them could not provide any information on the UVI. Even more problematic might be that about one-third (7 of 20) gave an incorrect definition. This leads to the discussion of two questions: How can pharmacists and pharmaceutical technicians be better trained, and is the UVI an instrument which is easy to understand and therefore useful in counselling pharmacy customers?

### 4.1. Training of Pharmacists and Pharmaceutical Technicians

We found that the term “UV index” was frequently confused with the term “sun protection factor”, an observation that has already been made in population surveys. In five repetitive cross-sectional studies between 2002 and 2008 in Switzerland, one-fifth of the respondents who stated to know the meaning of the UVI actually confused it with the SPF [40]. However, UVI and SPF describe very different concepts: While the UVI describes the actual intensity of UV radiation-producing sunburns on human skin at a specific place and time, the SPF is used for labelling sunscreen products. SPF is a measure of how much UV radiation is required to produce sunburn on protected skin (i.e., in the presence of sunscreen) relative to the amount of UV radiation required to produce sunburn on unprotected skin. As the SPF value increases, sunburn protection increases, while an increasing UVI corresponds to increasing sunburn risk. A reason for the confusion between UVI and SPF might be that the focus of training for pharmacists is on sunscreen use and counselling regarding the SPF, while the definition and implications of the UVI are considered less prominent in such training. If health organizations such as WHO aim to promote the use of the UVI to assist in the choice of suitable sun protection measures, health professionals who provide related counselling need more intensive training on this aspect, which implies necessary revisions to actual training curricula, at least in Germany.

### 4.2. Use of the UV Index during Counselling in Pharmacies

Participants reported that the term “UV index” was rarely used during counselling. Even those who could define it did not use the term itself but used it to make recommendations for the customers depending on where they planned to use sunscreen (e.g., during beach holidays). The fact that the term is not frequently used by pharmacists and pharmaceutical technicians may be due to the perceived complexity of the UVI. Many people are not aware of the term. In Germany, awareness of the UVI was found to be lower than 30% in the general population [41,42], and less than 10% of respondents were able to correctly define the term “UV index” [41]. Other studies in Germany restricted to specific subgroups of the population have found very similar results [43,44]. This lack of knowledge makes it almost impossible to use the term “UV index” during counselling in pharmacies.

To make the UVI a useful tool for improving sun protection behaviour in the short term, and for reducing skin cancer incidence in the long term, it needs to be better promoted not only within the general population but also among health professionals like pharmacists in Germany. The spread of this knowledge would also assist in making the UVI a helpful tool during counselling in pharmacies. One of the main obstacles in achieving better awareness, understanding, and use of the UVI in Germany is the lacking public exposure to the UVI. Currently, the availability of information about actual UVI levels and UVI forecasts for the general public in Germany is quite limited. Forecasts of the UVI are rarely incorporated into weather forecasts published in newspapers or broadcasted by TV channels (except on extreme days during summer). Some of the weather apps and specific apps for UVI monitoring like the showcase app “GlobalUV” developed for use on smartphones and tablets, however, include or even focus on UVI information. The information can also be found on specific websites operated by the German Weather Service and the Federal Office for Radiation Protection. Thus, informed people in Germany have the opportunity to get access to the information about the current UVI and its forecast, but it needs an active search behaviour to get the information and is therefore restricted to those who are already aware of the UVI and recognize the value of UVI information.

### 4.3. Limitations

We used our qualitative study to explore knowledge regarding the UVI in a specific population of pharmacy workers. This explorative approach was necessary to identify whether there are deficits in awareness, understanding, and use of the UVI. Based on this study, further research questions for future qualitative as well as quantitative projects can be developed. Nonetheless, there are some limitations that should be considered when interpreting our results. Our study used a qualitative design based on a convenient sample that may not be representative due to different reasons for non-participation (e.g., lack of time/no will to participate), social desirability, or potential fear of giving incorrect answers. This means our findings are not generalizable to all of Germany or other countries. However, generalizability and representativeness are not the aims of a qualitative approach. Instead, qualitative research aims to understand an individual’s attitudes, opinions, and beliefs. In addition, based on our findings, questions and item sets for quantitative studies can be developed to analyze the knowledge of pharmacists and pharmaceutical technicians in large samples. We tried to get a broad sample including pharmacists as well as pharmaceutical technicians. However, since knowledge in our sample was low, we were not able to stratify our findings. This implies also for potential subgroup analysis regarding sex, education, and attendance of specific sun protection training, that could not be performed due to our sample size. Future research should consider gaps in health professionals’ knowledge, as we did not expect this extent.

## 5. Conclusions

Our qualitative study of pharmacists and pharmaceutical technicians revealed severe deficits in awareness, understanding, and use of the UVI. Especially during customer counselling by pharmacists and pharmaceutical technicians, the UVI has the potential of being a useful tool to provide customers with information regarding appropriate sun protection. To achieve this goal, more information on the UVI and its use need to be provided (1) to those working in pharmacies during professional training, to ensure optimal counselling; and (2) to the general population to be more knowledgeable regarding UV radiation and be responsive to the implications of the UVI.

## Figures and Tables

**Table 1:** Characteristics of 20 pharmacists and pharmaceutical technicians participating in qualitative interviews.

ID	Age	Sex	Highest Vocational Qualification	Graduation Year	Position	Working Time	Advanced Training for Skin and Sun Protection
01	23	female	completed vocational training	2015	pharmaceutical technician	full-time	yes
02	60	female	master’s degree or doctorate	1984	salaried pharmacist	part-time	yes
03	62	male	master’s degree or doctorate	1982	holder/head	full-time	yes
04	54	female	completed vocational training	1987	pharmaceutical technician	part-time	yes
05	23	female	completed vocational training	2019	pharmaceutical technician	full-time	no
06	60	female	master’s degree or doctorate	1983	salaried pharmacist	part-time	no
07	58	male	master’s degree or doctorate	1985	holder/head	full-time	yes
08	48	female	completed vocational training	2001	pharmaceutical technician	full-time	yes
09	26	female	completed vocational training	2017	pharmaceutical technician	full-time	yes
10	58	female	master’s degree or doctorate	1985	salaried pharmacist	part-time	no
11	64	female	completed vocational training	1983	pharmaceutical technician	part-time	no
12	52	female	master’s degree or doctorate	1992	salaried pharmacist	full-time	yes
13	51	female	completed vocational training	1987	pharmaceutical technician	full-time	yes
14	28	female	completed vocational training	2014	pharmaceutical technician	part-time	no
15	29	female	completed vocational training	2013	pharmaceutical technician	full-time	yes
16	32	female	completed vocational training	2011	pharmaceutical technician	full-time	yes
17	47	male	master’s degree or doctorate	2000	holder/head	full-time	yes
18	53	male	master’s degree or doctorate	1991	holder/head	full-time	yes
19	69	female	master’s degree or doctorate	1980	salaried pharmacist	part-time	yes
20	53	female	master’s degree or doctorate	1992	salaried pharmacist	part-time	yes

**Table 2:** Incorrect definitions of the UV index given by pharmacists and pharmaceutical technicians.

P03	*“The UV index, um, is the classification of, um, the sun protection for UVA and UVB radiation, um, and we have, um, up to 50, normal, um, UVB index, if I understood that correctly. Yes, and there are special numbers, um, for the UVA assessment. That’s the case with the [company name] products, um, it’s called [name], um, [name] or something like that, I don’t know by heart now. But there is an abbreviation aimed at the UVA radiation which is more harmful.”*
P04	*“(..) it says how long you/how long the self-protection/how many times the self-protection is prolonged. That’s what I understand by UV index.”*
P05	*“I think UV index are these numbers, 30, 50, so this factor which is supposed to protect somehow, like more intensive, a little less, I would think it’s like that.”*
P08	*“The UV index, um, I’m not sure 100 per cent, has something to do with the fairness of the skin and the, um, time of exposure, I thought.”*
P09	*“Um, maybe how, or to what extent, how long a sun protection product actually protects the skin.”*
P11	*“(.) um, I think it means which radiation types are filtered and um, to compare the products which (…) label the protection.”*
P15	*“Yes, maybe it’s the number of minutes I can stay in the sun without being burnt. Or maybe the intensity of the sun or something like that, I don’t remember. But I’ve heard of it before.”*

**Table 3:** Answers regarding the values of UV index by pharmacists and pharmaceutical technicians.

Values of UV Index (General Minimum and Maximum Values)			
Answers with uncertainty		Answers without uncertainty	
P06	*“7–8 in this region. In the Mediterranean up to 12. Is that correct? (laughing)”*	P02	*“It’s a measurement for the intensity of the solar radiation, in Germany, it reaches from 1 to 11, but 11 isn’t really achieved, in Germany, we have 8 to 10, 10 maximum; last year, I was in Burundi, there was UV index 15 and I didn’t know that this value is possible, I thought the scale was 1 to 11. So there is also 15 and I had a sunburn within 6 min.”*
P12	*“Um, I think the scale reaches to 10 but I don’t know exactly.”*	P10	*“It’s about how intensive the sun rays are when they reach the Earth. It depends on the diameter of the ozone layer. This depends on which latitude you are right now and it also depends on the season, of course, and the values reach from 1 to 11 or 0 to 11 or something like that. The first three levels mean little strain. In the next few levels, the strain is medium and from 8 onwards it‘s dramatic. You have to be really careful then. Of course, it’s very important to not only use sunscreen then, but also hats, t-shirts etc.”*
P13	*“Oh, I think 10, right, yes.”*		
**Maximum values of UV index in Rhine-Neckar region**			
Answers with uncertainty		Answers without uncertainty	
P06	*“7–8 in this region. […] Is that correct? (laughing)”*	P02	*“8 to 10.”*
P10	*“Well, I think it can be 10, 11.”*		
P12	*“If you tell me afterwards if I am right with the scale from 1–10, then I’d say we are approximately in the center, that’s what I would estimate. We are not at the lower end but also not at the upper end.”*		
P13	*“I think we already had 6, the UV index has already been 6 when I look it up from time to time, but I don’t know if it can reach 7, but I don’t think it goes above that.”*		
**Value at Which Protection is Recommended**			
Answers with uncertainty		Answers without uncertainty	
P06	*“(..) um (.) yes, even if it’s low, there is always UV radiation. I estimate from 3 onwards.”* *“And above which value special protective measures are required?”* *“From 5 on, I suppose.”*	P02	*“From 3/3,4,5 on you should use sunscreen, if you are an especially sensitive person with a skin condition, actinic keratosis or something like that.“*
P10	*“Already from 4 onwards, I think.“* *“And above which value special protective measures are recommended?“* *“Seven.“*		
P13	*“[…] (laughing) from 2 onwards […]“* *“[…] And special protective measures, meaning more that the usual sun protection measures?“* *[…]* *“I would say above 6, 7.“*		

## Data Availability

The data presented in this study are available on request from the corresponding author.

## Supplementary Materials

The following are available online at https://www.mdpi.com/1660-4601/18/4/1615/s1, Interview guide.

## References

[1] Erdmann F., Lortet-Tieulent J., Schuz J., Zeeb H., Greinert R., Breitbart E.W., Bray F. (2013). International trends in the incidence of malignant melanoma 1953–2008—are recent generations at higher or lower risk?. Int. J. Cancer.

[2] Garbe C., Leiter U. (2009). Melanoma epidemiology and trends. Clin. Derm..

[3] Lomas A., Leonardi-Bee J., Bath-Hextall F. (2012). A systematic review of worldwide incidence of nonmelanoma skin cancer. Br. J. Derm..

[4] Matthews N.H., Li W.Q., Qureshi A.A., Weinstock M.A., Cho E., Ward W.H., Farma J.M. (2017). Epidemiology of melanoma. Cutaneous Melanoma: Etiology and Therapy.

[5] Nehal K.S., Bichakjian C.K. (2018). Update on keratinocyte carcinomas. N. Engl. J. Med..

[6] Armstrong B.K., Kricker A. (2001). The epidemiology of UV induced skin cancer. J. Photochem. Photobiol. B.

[7] El Ghissassi F., Baan R., Straif K., Grosse Y., Secretan B., Bouvard V., Benbrahim-Tallaa L., Guha N., Freeman C., Galichet L. (2009). A review of human carcinogens—Part D: Radiation. Lancet Oncol..

[8] Lucas R., McMichael T., Smith W., Armstrong B. (2006). Solar Ultraviolet Radiation: Global Burden of Disease from Solar Ultraviolet Radiation.

[9] Silva E.S.D., Tavares R., Paulitsch F.D.S., Zhang L. (2018). Use of sunscreen and risk of melanoma and non-melanoma skin cancer: A systematic review and meta-analysis. Eur. J. Derm..

[10] Gefeller O., Pfahlberg A. (2002). Sunscreen use and melanoma: A case of evidence-based prevention?. Photodermatol. Photoimmunol. Photomed..

[11] Berwick M. (2011). The good, the bad, and the ugly of sunscreens. Clin. Pharm..

[12] Görig T., Diehl K., Greinert R., Breitbart E.W., Schneider S. (2018). Prevalence of sun-protective behaviour and intentional sun tanning in German adolescents and adults: Results of a nationwide telephone survey. J. Eur. Acad Derm. Venereol..

[13] Mayer J.A., Eckhardt L., Stepanski B.M., Sallis J.F., Elder J.P., Slymen D.J., Creech L., Graf G., Palmer R.C., Rosenberg C. (1998). Promoting skin cancer prevention counseling by pharmacists. Am. J. Public Health.

[14] GfK (2016). Trust in Professions.

[15] Szabo L., Podlogar J. (2018). Sonnenschutz ist alles. Dtsch. Apoth. Ztg..

[16] Tucker R., Duffy J. (2014). The role of community pharmacists in the management of skin problems. J. Pharm. Care Health Syst..

[17] Uter W., Fiessler C., Gefeller O., Pfahlberg A. (2017). Kenntnisse und Wissensquellen zu Hautkrebs-Risikofaktoren bei Eltern von 3- bis 6-jährigen Kindern. Bundesgesundheitsblatt Gesundh. Gesundh..

[18] Schmalwieser A.W., Grobner J., Blumthaler M., Klotz B., De Backer H., Bolsee D., Werner R., Tomsic D., Metelka L., Eriksen P. (2017). UV Index monitoring in Europe. Photochem. Photobiol. Sci..

[19] Lehmann M., Sandmann H., Pfahlberg A.B., Uter W., Gefeller O. (2019). Erythemal UV radiation on days with low UV Index values—An analysis of data from the German Solar UV Monitoring Network over a ten-year period. Photochem. Photobiol..

[20] Blumthaler M. (2018). UV Monitoring for Public Health. Int. J. Environ. Res. Public Health.

[21] Commission Internationale de l’Eclairage (CIE) (1998). Erythema Reference Action Spectrum and Standard Erythema Dose. ISO 17166:1999(E)/CIE S 007/E-1998.

[22] Webb A.R., Slaper H., Koepke P., Schmalwieser A.W. (2011). Know your standard: Clarifying the CIE erythema action spectrum. Photochem. Photobiol..

[23] Fioletov V., Kerr J.B., Fergusson A. (2010). The UV index: Definition, distribution and factors affecting it. Can. J. Public Health.

[24] International Commission on Non-Ionizing Radiation Protection (1995). Global Solar UV Index—A Joint Recommendation of the WHO, WMO, UNEP and the ICNIRP.

[25] World Health Organization, World Meteorological Organization, United Nations Environment Programme, International Commission on Non-Ionizing Radiation Protection (2002). Global Solar UV Index. A Practical Guide.

[26] Allinson S., Asmuss M., Baldermann C., Bentzen J., Buller D., Gerber N., Green A.C., Greinert R., Kimlin M., Kunrath J. (2012). Validity and use of the UV index: Report from the UVI working group, Schloss Hohenkammer, Germany, 5–7 December 2011. Health Phys..

[27] Gies P., van Deventer E., Green A.C., Sinclair C., Tinker R. (2018). Review of the Global Solar UV Index 2015 Workshop Report. Health Phys..

[28] Lehmann M., Pfahlberg A.B., Sandmann H., Uter W., Gefeller O. (2019). Public health messages associated with low UV index values need reconsideration. Int. J. Environ. Res. Public Health.

[29] Vanicek K., Frei T., Litynska Z., Schmalwieser A.W. (2000). UV Index for the Public.

[30] Heckman C.J., Liang K., Riley M. (2019). Awareness, understanding, use, and impact of the UV index: A systematic review of over two decades of international research. Prev. Med..

[31] Italia N., Rehfuess E.A. (2012). Is the Global Solar UV Index an effective instrument for promoting sun protection? A systematic review. Health Educ. Res..

[32] Mayring P., Mey G., Mruck K. (2010). Qualitative Inhaltsanalyse. Handbuch Qualitative Forschung in der Psychologie.

[33] Brennan R.L., Prediger D.J. (1981). Coefficient kappa: Some uses, misuses, and alternatives. Educ. Psychol. Meas..

[34] Tong A., Sainsbury P., Craig J. (2007). Consolidated criteria for reporting qualitative research (COREQ): A 32-item checklist for interviews and focus groups. Int. J. Qual. Health Care.

[35] Schmiedel K., Schlager H., Dorje F. (2013). Preventive counselling for public health in pharmacies in South Germany. Int. J. Clin. Pharm.

[36] Almuqati R.R., Alamri A.S., Almuqati N.R. (2019). Knowledge, attitude, and practices toward sun exposure and use of sun protection among non-medical, female, university students in Saudi Arabia: A cross-sectional study. Int. J. Womens Derm..

[37] Armstrong E.P., Campbell C., Van Allen A., Vincent E. (2010). Skin cancer knowledge and prevention counseling among Arizona pharmacists. J. Pharm Pr..

[38] Ghiasi G., Hashemian F., Kebriaeezedeh A., Ghiasi S. (2016). The knowledge of pharmacists about cosmetics in pharmacies of Tehran, Iran. J. Pharm. Pharm. Manag..

[39] Mayer J.A., Slymen D.J., Eckhardt L., Rosenberg C., Stepanski B.M., Creech L., Palmer R.C., Elder J.P., Graf G., Anderson S.T. (1998). Skin cancer prevention counseling by pharmacists: Specific outcomes of an intervention trial. Cancer Detect. Prev..

[40] Krebs H. (2008). Bekanntheit und Bekanntmachung des UV-Index 2002–2008.

[41] Börner F.U., Schutz H., Wiedemann P. (2010). The influence of the UV-index on attitudes toward sun exposure in the German population. J. Cancer Educ..

[42] Capellaro M., Sturm D. (2015). Evaluation von Informationssystemen zu Klimawandel und Gesundheit Band 1: Anpassung an den Klimawandel: Evaluation bestehender nationaler Informationssysteme (UV-Index, Hitzewarnsystem, Pollenflug- und Ozonvorhersage) aus Gesundheitlicher Sicht—Wie Erreichen wir die Empfindlichen Bevölkerungsgruppen?.

[43] Klostermann S., Bolte G., Group G.M.E.S. (2014). Determinants of inadequate parental sun protection behaviour in their children--results of a cross-sectional study in Germany. Int. J. Hyg. Environ. Health.

[44] Hault K., Ronsch H., Beissert S., Knuschke P., Bauer A. (2016). Knowledge of outdoor workers on the effects of natural UV radiation and methods of protection against exposure. J. Eur. Acad. Derm. Venereol..

