# Supplementary material for: “I’ve Heard of It, Yes, but I Can’t Remember What Exactly It Was”—A Qualitative Study on Awareness, Knowledge, and Use of the UV Index
Source: Int J Environ Res Public Health. 2021 Feb 8;18(4):1615. doi: 10.3390/ijerph18041615 (PMC7914494; doi:10.3390/ijerph18041615)
Supplement: Supplementary file 1 [file ijerph-18-01615-s001.pdf]

Supplementary material:

Interview guide: Questions during the interview addressing the UV index

Question 1: Have you ever heard of the term UV index?

Question 2, alternative 1: What do you understand by the term UV index?
Question 2, alternative 2: Do you have any idea, what the term UV index means?

Question 3: Do you know which values the UV index can assume?
Further queries:

- Which range of values does the UV index have?

Question 4: What do you think, what are the maximum values of the UV index in our region in summer?

Question 5: What do you think is the value from which sun protection measures are recommended? What is the value from which special protection measures are necessary?

Question 6: If you know the current UV index value, does this information influence your behavior on the concerning day?
Further queries:

- How does this information influence your behavior?

Question 7: To which extent does the awareness of the current UV index value influence your counselling of customers in the pharmacy?

Question 8: To which extent do you share your knowledge about UV index with your customers?
